# Supplementary material for: Systemic HIV-1 infection produces a unique glial footprint in humanized mouse brains
Source: Dis Model Mech. 2017 Dec 1;10(12):1489–502. doi: 10.1242/dmm.031773 (PMC5769612; doi:10.1242/dmm.031773)
Supplement: Supplementary information [file dmm-10-031773-s1.pdf]

Supplementary information

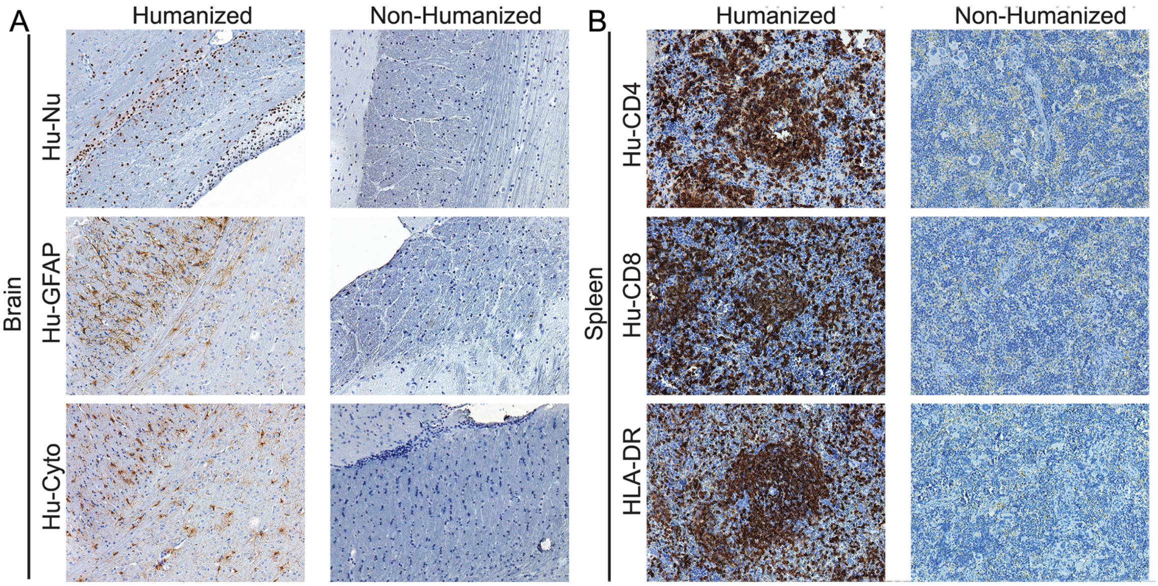

Figure S1. **Species-specificity of antibodies.** Humanized and non-humanized mice brain and spleen paraffin-embedded 5-μ-thick sections were stained with human-specific antibodies to confirm their species-specificity. Dako EnVision kit and and diaminobenzidene (DAB) were the detection system, brown staining was considered as positive. All sections were counterstained with Mayer's hematoxylin. (**A**) Representative humanized mice brain slides staining for human cells with antibodies to nuclear antigen (Hu-Nu, 1:100, Millipore/MAB4383, Billerica, MA, USA,), glial fibrillary acidic protein (Hu-GFAP, 1:1000; Y40420/STEM123, TaKaRa Bio, USA) and cytoplasmic marker (Hu-Cyto, 1:1000; Takara/STEM121, Mountain View, Canada). Non-humanized mice tissue sections were negative. (**B**) Spleen's sections from humanized and non-humanized mice were stained for HLA-DR (1:500; Dako/M0746, Carpinteria, CA, USA), CD4 and CD8 antigens (1:500; Abcam/ab133616 and ab101500). Positive staining was observed only in humanized mice sections. Images were captured by a Nuance multiplex system (CRi, Wobum, MA) with an original magnification of 200×.

Disease Models & Mechanisms • Supplementary information

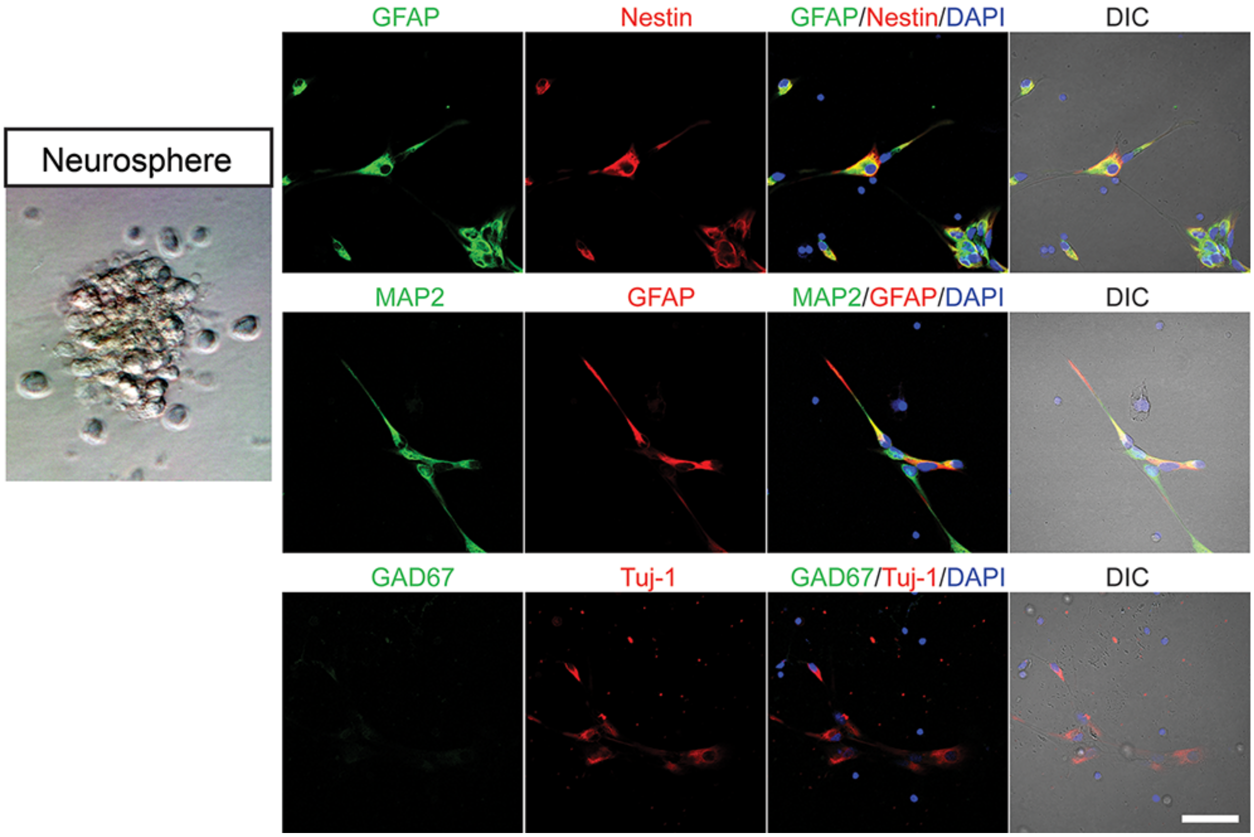

Figure S2. **Properties of NPC**. Example of neurosphere in culture. Image obtained by a Nikon TE300 microscope (Nikon Instruments, Inc., Melville, NY, USA) at original magnification of 200×. Representative immunofluorescent images of 48-hour cultured single cells derived from neurospheres. Upper line panels show cells positive for glial fibrillary acidic protein (GFAP, green) and nestin (red), both known as progenitor cells with radial glia characteristics. Middle line panels demonstrate cells stained by microtubule-associated protein 2 (MAP2, green) and GFAP (red) antibodies, which are considered to be early stage glial precursors. Lower line panels show cells positively stained by the neuronal marker class III β-tubulin (Tuj-1; red) and not glutamic acid decarboxylase 67 (GAD67; green). Original images were captured by a Zeiss LSM510 system (Carl Zeiss Microscopy GmbH, Jena, Germany) with 400× magnification. Bar = 50 µm.

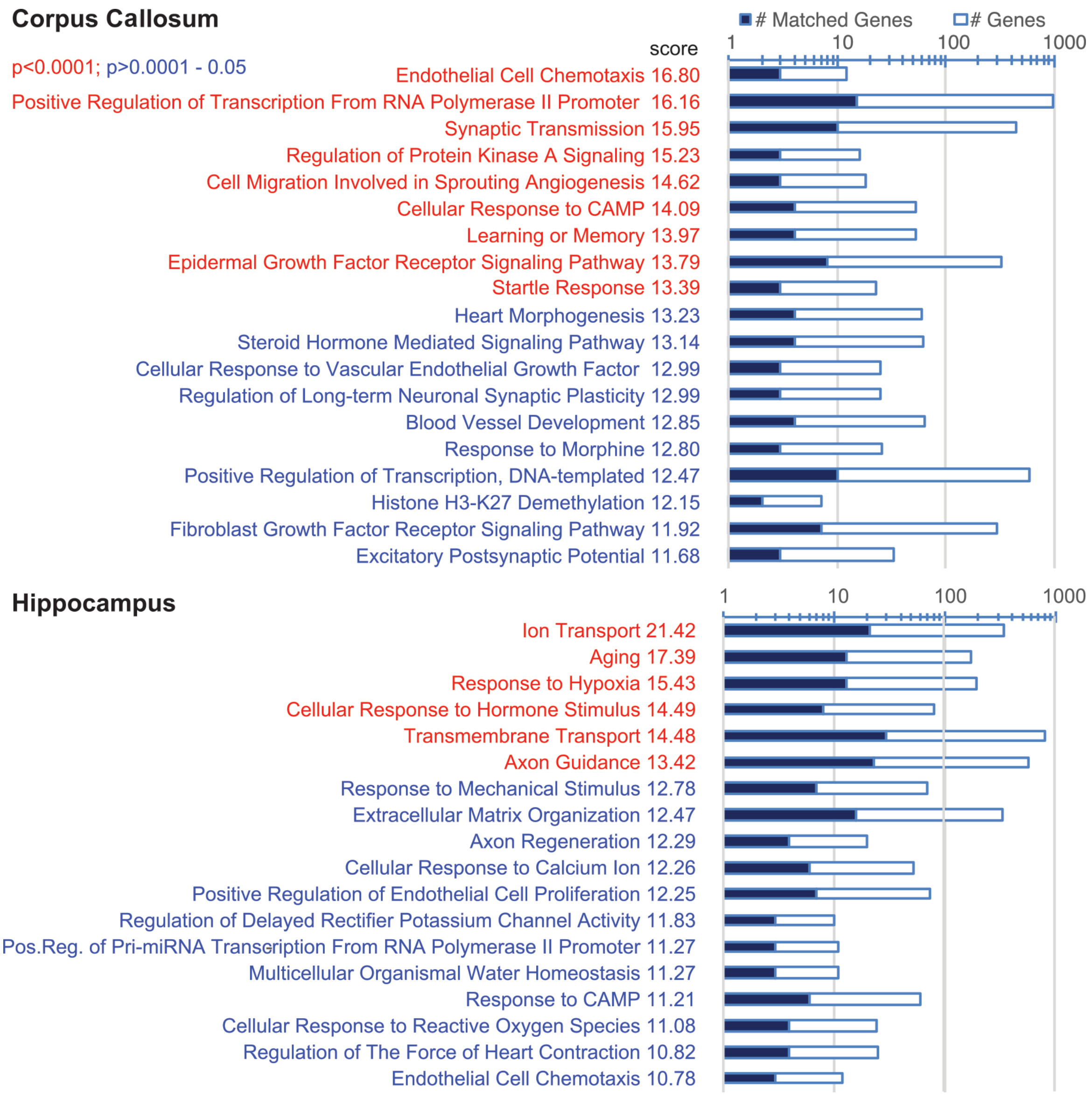

Figure S3. **Gene Ontology (GO) enrichment analysis for differentially expressed gene sets according to the mouse genome.** Results are reflected according to murine data base and predicted biologic processes. These processes are operative in the mouse brain associated with dual immune and brain humanization**.** The complete information including gene sets and FPKM expression are shown in the supplemental data present in Supplemental Table S1 and S2.

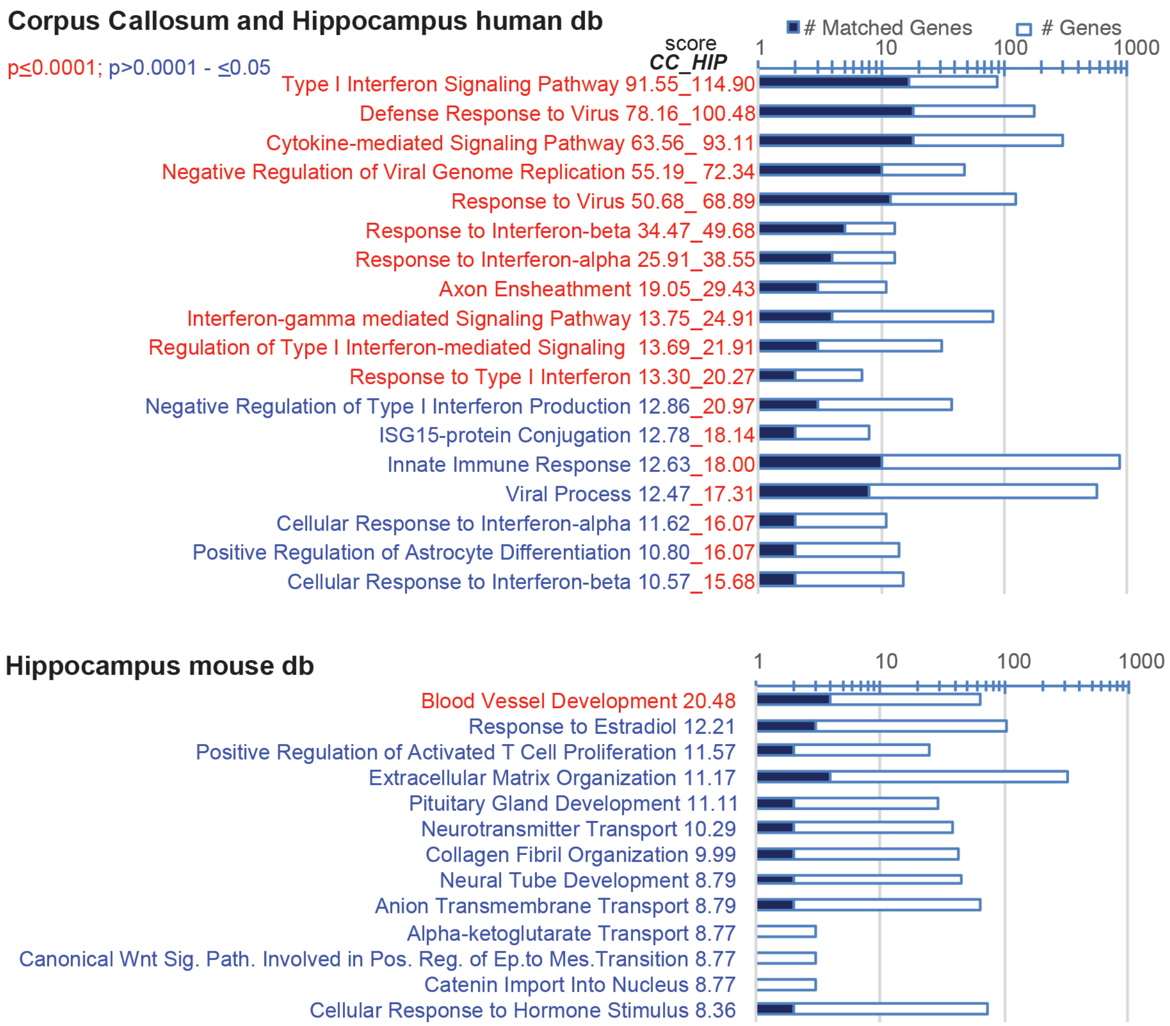

Figure S4. **Gene Ontology (GO) enrichment analysis for gene sets identified as differentially expressed according to human and mouse data bases.** These were observed in HIV-1 infected humanized mice and affect the listed biologic processes. There is overlap for identified human genes for Hip and CC (hg19) and different biological processes were predicted for mouse transcriptome (mm10). The complete information including gene sets and analysis are present in the supplemental data sets present in Supplemental Tables S1 and S2.

Disease Models & Mechanisms • Supplementary information

Table S1. Transcriptional changes induced by human NPC transplantation evaluated at 6 months of age (mm10).

Click here to Download Table S1

Table S2. HIV-1 effects on transcriptional profile of human and mouse genes and suggested biologic processes.

Click here to Download Table S2

Disease Models & Mechanisms • Supplementary information
